# Metabolomics of Dynamic Changes in Insulin Resistance Before and After Exercise in PCOS

**DOI:** 10.3389/fendo.2019.00116

**Published:** 2019-02-27

**Authors:** Anna Halama, Myint Myint Aye, Soha R. Dargham, Michal Kulinski, Karsten Suhre, Stephen L. Atkin

**Affiliations:** ^1^Department of Physiology and Biophysics, Weill Cornell Medicine-Qatar, Doha, Qatar; ^2^Department of Academic Endocrinology, Diabetes and Metabolism, Hull York Medical School, Hull, United Kingdom; ^3^Infectious Disease Epidemiology Group, Weill Cornell Medicine, Doha, Qatar; ^4^Translational Research Institute, Academic Health System, Hamad Medical Corporation, Doha, Qatar; ^5^Weill Cornell Medicine-Qatar, Doha, Qatar

**Keywords:** insulin resistance, intralipid, exercise, PCOS, metabolomics, amino acid

## Abstract

**Background:** Plasma elevated levels of branched chain amino acids (BCAA) and aromatic amino acids (AAA) have been associated with obesity and insulin resistance, but their relationship to stimulated insulin resistance (IR) in PCOS and in response to exercise is unknown. Indeed, it is unknown whether the mechanism of IR in PCOS is mediated through changes in the metabolome.

**Methods:** Twelve women with polycystic ovary syndrome (PCOS) and ten age and body mass index matched controls completed an 8 week supervised exercise program at 60% maximal oxygen consumption. Before and after the exercise program, all participants underwent maximal IR stimulation with intralipid infusions followed by insulin sensitivity (IS) measurement by hyperinsulinaemic euglycaemic clamps. Amino acid profiles and metabolites were taken at baseline and at maximal insulin resistance stimulation before and after the exercise program.

**Results:** At baseline, PCOS subjects showed increased leucine/isoleucine, glutamate, methionine, ornithine, phenylalanine, tyrosine and proline (*p* < 0.05) that, following exercise, did not differ from controls. While compering within the groups, no significant changes in the amino acid levels before and after exercise were observed. Exercise improved VO2 max (*p* < 0.01) but did not alter weight. Amino acid profiles were unaffected by an acute increase in IR induced by the lipid infusion. IS was lower in PCOS (*p* < 0.001) and was further decreased by the lipid infusion in both PCOS and controls. Although, exercise improved IS in both PCOS and in controls, the IS remained compromised in PCOS.

**Conclusion:** The baseline amino acid profile in PCOS reflected that seen in obese subjects and differed to controls. After exercise, and despite no change in weight in either group, there were no differences in the amino acid profile between PCOS and controls. This shows that exercise may normalize the amino acid metabolome, irrespective of weight.

**ISRCTN number**: ISRCTN42448814

## Introduction

Polycystic ovary syndrome (PCOS) is one of the most common endocrine disorders, affecting up to 20% of reproductive-aged women (1, 2). There are three available diagnostic criteria for PCOS; the National Institute of Health (NIH) (3), ESHRE/ASRM Rotterdam consensus criteria (4) and the androgen excess and PCOS society (AES) (5). However, by any of the diagnostic criteria, PCOS is associated with metabolic features commonly related to lower insulin sensitivity (IS) that is contributed to by an increased BMI in women with PCOS compared to those without PCOS (6); PCOS subjects that were overweight showed moderately lower IS compared with lean PCOS (7). This is reflected in an increased prevalence of gestational diabetes, impaired glucose tolerance and type 2 diabetes (5-fold in Asia, 4-fold in the Americas, and 3-fold in Europe) in PCOS irrespective of age, with risk independent of, though exacerbated by, obesity (2).

The strategies addressing health issues in women with PCOS mainly focus on the reproductive dysfunction and compromised sensitivity to insulin [insulin resistance (IR)] (8). It has been shown that physical activity significantly reduced IR and improved lipid homeostasis in obese individuals at risk of developing type 2 diabetes (T2D) (9). There is also increasing evidence suggesting that moderate exercises could be considered as a strategy to reduce IR in PCOS patients as a significant improvement in IS (23–30%) was observed in several studies involving PCOS patients before and after exercise (10–12). Normalization of insulin responses in patients is crucial for metabolic homeostasis and thus body function. Moderate intensity exercise increases mitochondrial fatty acid oxidation and decreases diacylglycerol (DAG) and ceramide content of skeletal muscle in obese subjects (13); remarkably, a single session of exercise completely normalized fatty acid accumulation and reversed fatty acid-induced IR in healthy subjects (14).

The bioanalytical discipline of metabolomics measures the small molecules (metabolites) that are mediators and products of metabolism that has been shown to provide further insights into the field of complex diseases (15). For instance, metabolomics based studies have revealed an association between IR and deregulated metabolism of aromatic amino acids (AAA) such as phenylalanine and tyrosine (16) as well as branched chain amino acids (BCAA) (isoleucine, valine and leucine), and different carnitines (16–18). The interplay between BCAA and lipids was suggested as a contributor to the development of IR in obese individuals (19). Thus, it could be hypothesize that the moderate exercise might improve IR and thus overall body metabolism in PCOS patients.

The complex metabolic pathways underlying PCOS are not well understood though the few studies focused on describing deregulated carbohydrate, fat and protein metabolism have been conducted (20–24). However, to the best of our knowledge the impact of moderate exercise on overall body metabolism in PCOS patients, has not been investigated. Given that IR was shown to be associated with deregulated BCAA and AA metabolism (17), we hypothesized that moderate exercise would decrease the IR in PCOS and thus improve the overall metabolic status.

Lipid infusion delivers a dynamic and maximal measure of IR, and the gold standard for IR measurement is the hyperglycemic glucose clamp method to determine IR change with an intervention. An acute fat load orally or intravenously results in an acute rise in DAG in the muscle that subsequently induces insulin resistance by reducing non-oxidative glucose disposal through protein kinase C theta (PKCθ) activation (25).

The aim of our study was to investigate the overall metabolic homeostasis after 8 weeks of moderate exercise in women with and without PCOS in the context of IR. The metabolic parameters were investigated before and after exercise under conditions of 1, hyperinsulinaemic euglycaemic clamp and saline infusions as well as 2, hyperinsulinaemic euglycaemic clamp and intralipid infusions to exaggerate the metabolic responses.

## Subjects and Methods

This study was approved by the Leeds Central Research Ethics Committee, Yorkshire and Humber REC no. 10/H1313/44 and all subjects gave their written informed consent to participate in the study. Subjects with PCOS were recruited from endocrine clinics, and normal female controls were recruited through advertisements at a local university and in the hospital newsletters. PCOS was diagnosed based on the presence of two out of three criteria; oligomenorrhoea, clinical or biochemical hyperandrogenism and polycystic ovaries on ultrasound after exclusion of other endocrine causes of hyperandrogenism according to the Rotterdam criteria (4); however, all PCOS women included in the study fulfilled all 3 criteria. All the subjects gave their informed consent to participate in the study. They were all non-smokers, took no regular medications, had no concurrent illness and had had no regular exercise prior to the study. All women had a pregnancy test prior to their inclusion in the study. Subjects who were diagnosed with impaired glucose tolerance at screening oral glucose tolerance test were excluded. Subjects were requested not to modify other aspects of their lifestyle, including their dietary pattern, during the study period. All the subjects had anthropometric measurements and fasting blood sampling. To assess maximal stimulation of insulin resistance, each subject had either 3 h saline or intralipid infusions at baseline; this was immediately followed by hyperinsulinaemic euglycaemic clamps with continuance of the saline or lipid infusion to assess insulin sensitivity. Normal control women had the initial clamp in the first week of their menstrual cycle, whilst PCOS women were clamped after 6 weeks of amenorrhea. These tests were repeated after completion of an 8 week moderate intensity exercise program.

### Hyperinsulinaemic Euglycaemic Clamps

Following a 12 h overnight fast, participants underwent a hyperinsulinaemic euglycaemic clamp that measured insulin sensitivity whilst receiving an infusion of normal saline on the first occasion, followed by a second intralipid infusion within a week. An 18 gauge intravenous cannula was inserted into an antecubital vein to administer test infusions and a retrograde cannula was inserted into the dorsal hand vein on the contralateral hand. This hand was heated (60°C) to arterialize venous blood for the measurement of blood glucose. On the lipid infusion day, an additional cannula was placed in the contralateral antecubital vein for the lipid emulsion. After fasted blood samples were taken, either normal saline 1.5 mL/min or 20% intralipid 1.5 mL/min, along with unfractionated heparin sodium 0.3 unit/kg/min, was infused for 5 h. At 180 min, a 2 h hyperinsulinaemic euglycaemic clamp was started using intravenous soluble insulin (Humulin S, Eli Lilly and Co., Indianapolis, IN) at a rate of 80 mU/m^2^ surface area/min for the first 20 min followed by a constant rate of 40 mU/m^2^ surface area/min for the remaining 100 min. Plasma glucose was clamped at 5.0 mmol/L with a variable infusion rate of 20% dextrose, adjusted relative to arterialized blood glucose measurements undertaken every 5 min. Endogenous glucose production was more than 90% suppressed by an acute rise of insulin with the primed insulin infusion (26). The rate of insulin stimulated glucose disposal was based on total body weight (mg/kg/min) (M value), a measure of insulin sensitivity, was calculated from the mean of the five 20 min periods from 20–120 min during the clamp using the Defronzo method (27). Blood samples were taken at baseline and every hour for 5 h. These samples were centrifuged at 1500 G for 15 min at 4°C within 15 min of sampling, and then plasma and serum stored at−80°C until analysis.

### Exercise Intervention

All participants underwent a structured supervised exercise program for a 1 h session, three times per week for 8 weeks in the sports laboratory at the Department of Sports, Health and Exercise Science, University of Hull. Moderate intensity exercise was defined as achieving a targeted heart rate equivalent to 60% of their baseline VO_2_ max. Measurement of individualized VO_2_ max was performed on a motorized treadmill starting with a warm up, then speeding to walking pace, then increasing every minute until the subject could not keep pace with the treadmill or became too tired to continue. Inspired and expired gas fractions, and heart rate were continuously monitored (28). All supervised exercise sessions achieved a targeted heart rate equivalent to 60% of their baseline VO_2_ max. A mid-point reassessment was conducted to adjust the moderate intensity based on improvements in exercise capacity.

### Biochemical Analysis

Serum insulin was assayed using a competitive chemiluminescent immunoassay (Euro/DPC, Llanberis, UK). Plasma glucose was measured using a Synchon LX 20 analyzer (Beckman-Coulter, High Wycombe, UK). Serum testosterone was measured by high performance liquid chromatography linked to tandem mass spectrometry (Waters Corporation, Manchester, UK) and sex hormone binding globulin (SHBG) was measured by immunometric assay with fluorescence detection on the DPC Immulite 2000 analyzer. The free androgen index (FAI) was obtained as the quotient 100^*^ Testosterone/SHBG. Total cholesterol, triglycerides (TG), and high density lipoprotein cholesterol (HDL-c) were measured enzymatically using a Synchon LX20 analyzer (Beckman-Coulter, High Wycombe, UK). LDL-c was calculated using the Friedewald equation (29).

### Metabolomics Measurements

For metabolomics analysis, samples were collected at (1) baseline; (2) 180 min after the saline/lipid infusion was started and (3) 120 min after the hyperinsulinaemic euglycaemic clamp was undertaken during the saline/lipid infusion (300 min from baseline). In total 288 samples were submitted out of which 7 were excluded due to insufficient volume of plasma. The remaining 281 samples consist of 131 controls and 150 PCOS.

The metabolites were measured in the Translational Research Institute (iTRI) at Hamad Bin Khalifa Medical City in Qatar using a targeted metabolomics assay from Biocrates Life Sciences AG (Innsbruck, Austria). The AbsoluteIDQ p150 kit assay enables quantification of up to 163 molecules including amino acids (AAs), phospatidylcholines (PCs), lysoPCs, sphingomyelines (SM), carnitines (Cs), and hexoses (H), as previously described (30, 31). Briefly, sample preparation and measurement was conducted in a 96-well plate. Ten microliter of plasma was applied on the AbsoluteIDQ p150 kit plate consisting of filters with internal standards. The sample underwent drying under a nitrogen stream at room temperature (RT). Dried samples were derivatized with derivatization reagent (20 μL per well) containing 5% phenylisothiocyanate (PITC) followed by drying under a nitrogen stream at RT. The sample metabolite and internal standards were extracted with 300 μL of 5 mM ammonium acetate in methanol. Next, samples were filtered by centrifugation, and 20 μL of the flow-through was transferred to the fresh deep, 96-well plate and diluted with 380 μL of running solvent. The plate was covered with the silicone mat, mixed and placed in the autosampler Eksigent Ultra LC 100 (SCIEX). The metabolite detection was performed by FIA-MS/MS on an QTRAP®/Triple Quad™ 4500 LC-MS/MS system (SCIEX). Data evaluation was performed with MetIDQ software package. The complete analytical process was performed using the MetIQ software package, which is an integral part of the AbsoluteIDQ kit. The internal standards processed together with the samples were used as reference for the calculation of metabolite concentrations. The metabolite concentrations are reported in μmol/l. The leucine is depicted as xleucine and is representing the amount of leucine and isoleucine as they cannot be depicted as separate components within this method.

### Data Analysis

Homeostatic model assessment of insulin resistance (HOMA-IR) was calculated by the formula: HOMA1-IR = fasting plasma insulin (μU/ml) × fasting plasma glucose (mmol/L)/22.5 (32).

Statistical analysis was performed using SPSS version 23.0 (SPSS Inc., Chicago, IL). Wilcoxon signed ranks test was applied to skewed variables that violated the assumptions of normality when tested with the Kolmogorov–Smirnov test, and paired sample *t*-test for normally distributed data within the group. Log transformation and then an unpaired *t*-test were undertaken on those data that were skewed. Data are presented as means ± SEMs. For all analyses, a two-tailed *P* ≤ 0.05 was considered to indicate statistical significance.

The Orthogonal Projections to Latent Structures (OPLS) analysis using time and saline/lipid challenge as phenotype was performed with SIMCA version 15 (Umetrics, Umea, Sweden).

## Results

Twelve women with PCOS and ten healthy subjects, comparable in terms of age and BMI, completed the study. Baseline characteristics of the subjects are summarized in Table 1. Subjects with PCOS had a significantly larger waist hip ratio (*p* = 0.02), higher FAI (*p* = 2.1 × 10^−3^), and HOMA-IR (*p* = 0.04), as well as lower HDL-c, and sex hormone binding globulin (SHBG) (*p* = 3.2 × 10^−3^) than controls. PCOS subjects were less physically fit, as we observed significantly lower level of VO_2_ max than in controls (*p* = 5.6 × 10^−3^).

**Table 1:** Baseline characteristics of participants.

	**Controls (*n* = 10)**	**PCOS (*n* = 12)**	***p*-value**
Age (year)	25.26 ± 2.0	28.27 ± 1.9	0.03^*^
BMI (kg/m2)	26.8 ± 2.0	29.4 ± 1.6	0.07
Waist (cm)	81.1 ± 4.5	98.8 ± 4.6	0.02^*^
WHR	0.79 ± 0.02	0.87 ± 0.02	0.15
Testosterone (nmol/L)	1.05 ± 0.11	1.51 ± 0.23	0.06
FAI	2.1 ± 0.65	6.77 ± 0.84	2.1 × 10^−3*^
SHBG (nmol/L)	69.6 ± 9.4	26 ± 5.1	3.2 × 10^−3*^
TC (mmol/L)	4.61 ± 0.24	4.13 ± 0.19	0.27
TG (mmol/L)	0.84 ± 0.06	1.25 ± 0.21	0.18
HDL-c (mmol/L)	1.48 ± 0.15	1.12 ± 0.06	0.14
LDL-c (mmol/L)	2.66 ± 0.18	2.33 ± 0.15	0.44
FPG (mmol/L)	4.89 ± 0.18	4.94 ± 0.16	0.97
HbA1c (mmol/mmoL)	33 ± 1.8	34 ± 0.84	1
TSH (iu/L)	1.9 ± 0.29	1.6 ± 0.17	0.59
HOMA-IR	1.34 (0.80, 2.13)	2.3 (1.3, 3.9)	0.04^*^
VO2 max (ml/kg/min)	36.3 ± 2.02	26.9 ± 1.40	5.6 × 10^−3*^

*Skewed variables are provided as the medians (25th, 75th percentile). Normally distributed variables are shown as the means ± SEM*.

* *Significantly relevant data*.

### Amino Acid Differences Between PCOS and Control

We quantified 163 metabolites after the lipid/saline challenges in PCOS and controls before and after exercise at three different time points [baseline (T1), 180 min after lipid/saline infusion (T2) and 120 min after the hyperinsulinaemic euglycaemic clamp was undertaken during the saline/lipid infusion (300 min from baseline) (T3)] using the targeted metabolomics assay based Biocrates AbsoluteIDQ p150 kit. The OPLS analysis revealed two clusters separating samples based on the lipid/saline infusion. Additionally, we observed clear separation between time point T2 and T3 (Supplementary Figure 1A). Long and short chain carnitines including octadecadienylcarnitine (C18:2), hexadecadienoylcarnitine (C16:2), tetradecadienoylcarnitine (C14:2), 2-methylbutyrylcarnitine (C5), propionylcarnitine (C3), and acetyl carnitine (C2) together with amino acids (methionine, tryptophan, and phenylalanine) contributed mainly to the separation between lipid and saline infusion as shown by the loading plot of the OPLS (Supplementary Figure 1B). The separation between PCOS and control was not observed on the OPLS plot (Supplementary Figure 2).

We further evaluated levels of amino acids (arginine, glutamate, histine, methionine, glycine, proline, serine, ornithine, phenylalanine, threonine, trypophan, tyrosine, valine, xleucine), and carnitines (C2-carnitine, C3-carnitine, C5-carnitine).

At baseline, before the exercise PCOS subjects showed increased levels of xleucine, glutamate, methionine, ornithine, phenylalanine, tyrosine, and proline (*p* < 0.05) (Table 2). The level of acylcarinitines including C2-carnitine, C3-carnitine, and C5-carnitine were not significantly different between PCOS and control (Table 2). We further monitored whether lipid infusion is significantly impacting the selected metabolites. We observed that the acute IR induced by lipid infusion is resulting in significant decrease in tryptophan level and increase C2-carnitine level in both control and PCOS. Additionally in PCOS group lipid infusion resulted in significant decrease in the levels of methionine and phenylalanine (Supplementary Table 1). While comparing the metabolic profiles of PCOS and controls under lipid infusion before exercises we observed that only proline remained significantly lower in PCOS and the other metabolites, differentiating PCOS and controls at baseline were not significant (Table 3).

**Table 2:** Differences in amino acids levels before and after exercise between PCOS and healthy controls.

	**Before exercise**			**After exercise**		
	**Control**	**PCOS**	***P*-value**	**Control**	**PCOS**	***P*-value**
	**Mean (*****SD*****)**	**Mean (*****SD*****)**		**Mean (*****SD*****)**	**Mean (*****SD*****)**	
Arginine	77.85 (16.58)	80 (17.63)	0.66	83.8 (18.32)	78.96 (17.76)	0.39
Glutamate	524.08 (61.14)	607.64 (102.77)	< 0.01	557.1 (94.16)	587.05 (102.84)	0.33
Histine	75.31 (12.23)	75.79 (9.06)	0.87	79.96 (13.81)	78.93 (10.98)	0.79
Methionine	28.68 (3.91)	33.74 (5.55)	< 0.001	31.16 (7.07)	34.07 (6.14)	0.16
Ornithine	44.78 (10.45)	54.98 (17.86)	0.01	46.93 (13.11)	52.33 (22.18)	0.34
Phenylalanine	48.95 (6.78)	53.66 (8.84)	0.04	52.16 (10.87)	56.44 (10.95)	0.21
Threonine	113.25 (29.02)	128.24 (28.43)	0.07	122.62 (36.26)	140.01 (39.74)	0.15
Trypophan	66.4 (7.83)	70.57 (9.9)	0.1	68.78 (12.16)	70.48 (8.69)	0.6
Tyrosine	55.58 (12.1)	66.91 (17.03)	0.01	58.33 (14.78)	65.64 (20.97)	0.2
Valine	203.88 (44.51)	227.89 (56.71)	0.1	223.05 (52.65)	234.73 (65.94)	0.53
xLeucine	164.29 (31.61)	185.96 (38.23)	0.03	180.9 (47.16)	196.23 (51.49)	0.32
C2acylcarnitine	4.603 (1.271)	4.71 (1.216)	0.76	4.787 (1.212)	5.033 (1.647)	0.59
C3acylcarnitine	0.317 (0.061)	0.341 (0.084)	0.24	0.327 (0.062)	0.349 (0.084)	0.33
C5acylcarnitine	0.123 (0.129)	0.117 (0.124)	0.33	0.123 (0.107)	0.126 (0.118)	0.52
**MEDIAN (RANGE)**						
Glycine	240.5 (246.0)	273.0 (655.0)	0.32	228.0 (387.0)	270.0 (240.0)	0.37
Proline	120.5 (133.3)	144.5 (160.8)	0.05	127.0 (133.0)	157.5 (169.1)	0.31
Serine	94.5 (82.4)	95.0 (115.6)	0.52	93.7 (100.2)	100.5 (65.0)	0.6

**Table 3:** Differences in amino acids levels before and after exercise between PCOS and healthy controls under acute IR induced by lipid infusion.

	**Before exercise**			**After exercise**		
	**Control**	**PCOS**	***P*-value**	**Control**	**PCOS**	***P*-value**
	**Mean (*****SD*****)**	**Mean (*****SD*****)**		**Mean (*****SD*****)**	**Mean (*****SD*****)**	
Arg	0.94 (0.15)	0.9 (0.17)	0.498	0.85 (0.14)	0.97 (0.15)	0.08
Gln	1.06 (0.12)	1.01 (0.12)	0.236	1.01 (0.17)	1.06 (0.16)	0.464
Gly	0.89 (0.09)	0.82 (0.12)	0.103	0.83 (0.12)	0.85 (0.12)	0.677
His	0.96 (0.11)	0.9 (0.12)	0.263	0.89 (0.16)	0.92 (0.11)	0.66
Met	0.82 (0.10)	0.74 (0.12)	0.110	0.74 (0.14)	0.83 (0.15)	0.173
Orn	0.95 (0.21)	0.87 (0.17)	0.323	0.88 (0.16)	0.85 (0.20)	0.727
Phe	0.84 (0.09)	0.79 (0.11)	0.192	0.78 (0.10)	0.84 (0.13)	0.306
Pro	0.91 (0.09)	0.83 (0.08)	0.015	0.82 (0.09)	0.84 (0.12)	0.792
Ser	0.93 (0.09)	0.87 (0.12)	0.164	0.89 (0.16)	0.89 (0.11)	0.994
Thr	0.90 (0.08)	0.86 (0.13)	0.365	0.84 (0.11)	0.87 (0.12)	0.482
Trp	0.64 (0.12)	0.64 (0.08)	0.885	0.61 (0.11)	0.70 (0.11)	0.071
Tyr	0.80 (0.07)	0.76 (0.11)	0.199	0.76 (0.09)	0.82 (0.14)	0.254
Val	0.96 (0.09)	0.9 (0.14)	0.179	0.89 (0.13)	0.94 (0.14)	0.406
xLeu	0.93 (0.10)	0.85 (0.15)	0.126	0.82 (0.12)	0.91 (0.16)	0.174

Next, we examined the impact of exercise on the metabolic profile at baseline and under acute IR induced by the lipid infusion. Although at the baseline there was no significant alteration before and after exercise in PCOS and control (Table 4), the significant differences in the levels of amino acids seen between PCOS and controls were no longer apparent after exercise (Table 2), suggesting normalization of the overall metabolic status. Under lipid infusion, we observed a significant decrease in the level of xleucine in control but not in PCOS subjects (Table 5), but there were no differences between control and PCOS subjects in the amino acid levels after exercise (Table 3).

**Table 4:** Assesment of amino acid differencess in PCOS and healthy controls before and after exercise.

	**Control**			**PCOS**		
	**Before exercise**	**After exercise**	***P*-value**	**Before exercise**	**After exercise**	***P*-value**
	**Mean (*****SD*****)**	**Mean (*****SD*****)**		**Mean (*****SD*****)**	**Mean (*****SD*****)**	
Arginine	77.85 (16.58)	83.8 (18.32)	0.265	80 (17.63)	78.96 (17.76)	0.838
Glutamate	524.08 (61.14)	557.1 (94.16)	0.169	607.64 (102.77)	587.05 (102.84)	0.485
Histine	75.31 (12.23)	79.96 (13.81)	0.243	75.79 (9.06)	78.93 (10.98)	0.273
Methionine	28.68 (3.91)	31.16 (7.07)	0.173	33.74 (5.55)	34.07 (6.14)	0.843
Ornithine	44.78 (10.45)	46.93 (13.11)	0.55	54.98 (17.86)	52.33 (22.18)	0.642
Phenylalanine	48.95 (6.78)	52.16 (10.87)	0.24	53.66 (8.84)	56.44 (10.95)	0.325
Threonine	113.25 (29.02)	122.62 (36.26)	0.347	128.24 (28.43)	140.01 (39.74)	0.248
Trypophan	66.4 (7.83)	68.78 (12.16)	0.438	70.57 (9.9)	70.48 (8.69)	0.974
Tyrosine	55.58 (12.1)	58.33 (14.78)	0.501	66.91 (17.03)	65.64 (20.97)	0.814
Valine	203.88 (44.51)	223.05 (52.65)	0.197	227.89 (56.71)	234.73 (65.94)	0.695
xLeucine	164.29 (31.61)	180.9 (47.16)	0.171	185.96 (38.23)	196.23 (51.49)	0.422
C2acylcarnitine	4.603 (1.271)	4.787 (1.212)	0.629	4.71 (1.216)	5.033 (1.647)	0.429
C3acylcarnitine	0.317 (0.061)	0.327 (0.062)	0.597	0.341 (0.084)	0.349 (0.084)	0.736
C5acylcarnitine	0.123 (0.129)	0.123 (0.107)	0.636	0.117 (0.124)	0.126 (0.118)	0.292
**MEDIAN (RANGE)**						
Glycine	240.5 (246.0)	228.0 (387.0)	0.860	273.0 (655.0)	270.0 (240.0)	0.93
Proline	120.5 (133.3)	127.0 (133.0)	0.125	144.5 (160.8)	157.5 (169.1)	0.333
Serine	94.5 (82.4)	93.7 (100.2)	0.502	95.0 (115.6)	100.5 (65.0)	0.369

**Table 5:** Assesment of amino acid differencess in PCOS and healthy controls before and after exercise under acute IR induced by lipid infusion.

	**Control**			**PCOS**		
	**Before exercise**	**After exercise**	***P*-value**	**Before exercise**	**After exercise**	***P*-value**
	**Mean (*****SD*****)**	**Mean (*****SD*****)**		**Mean (*****SD*****)**	**Mean (*****SD*****)**
Arg	0.94 (0.15)	0.85 (0.14)	0.149	0.9 (0.17)	0.97 (0.15)	0.305
Gln	1.06 (0.12)	1.01 (0.17)	0.393	1.01 (0.12)	1.06 (0.16)	0.316
Gly	0.89 (0.09)	0.83 (0.12)	0.161	0.82 (0.12)	0.85 (0.12)	0.574
His	0.96 (0.11)	0.89 (0.16)	0.270	0.9 (0.12)	0.92 (0.11)	0.763
Met	0.82 (0.10)	0.74 (0.14)	0.142	0.74 (0.12)	0.83 (0.15)	0.131
Orn	0.95 (0.21)	0.88 (0.16)	0.381	0.87 (0.17)	0.85 (0.20)	0.718
Phe	0.84 (0.09)	0.78 (0.10)	0.147	0.79 (0.11)	0.84 (0.13)	0.349
Pro	0.91 (0.09)	0.82 (0.09)	0.031	0.83 (0.08)	0.84 (0.12)	0.775
Ser	0.93 (0.09)	0.89 (0.16)	0.428	0.87 (0.12)	0.89 (0.11)	0.711
Thr	0.90 (0.08)	0.84 (0.11)	0.152	0.86 (0.13)	0.87 (0.12)	0.773
Trp	0.64 (0.12)	0.61 (0.11)	0.500	0.64 (0.08)	0.70 (0.11)	0.099
Tyr	0.80 (0.07)	0.76 (0.09)	0.195	0.76 (0.11)	0.82 (0.14)	0.213
Val	0.96 (0.09)	0.89 (0.13)	0.190	0.9 (0.14)	0.94 (0.14)	0.413
xLeu	0.93 (0.10)	0.82 (0.12)	0.031	0.85 (0.15)	0.91 (0.16)	0.352

### Effect of Lipid on Insulin Resistance Before and After Exercise Intervention

During the saline infusion, PCOS subjects had a lower rate of insulin stimulated glucose disposal compared to controls (3.25 ± 0.76 vs. 4.8 ± 1.64 mg/kg/min, *p* = 0.01). Lipid caused a reduction in the insulin stimulated glucose disposal rate in PCOS from 3.25 ± 0.76 to 1.14 ± 0.78 mg/kg/min, *p* = 1.9 × 10^−7^, and in controls from 4.8 ± 1.64 to 2.5 ± 1.54 mg/kg/min, *p* = 5.4 × 10^−4^ (Supplementary Table 2). After 8 weeks of moderate intensity exercise, there were concomitant rises in insulin sensitivity in PCOS patients (glucose disposal mg/kg/min: PCOS pre-exercise 3.25 ± 0.76; post-exercise 3.95 ± 1.45; *p* = 0.13), and in controls (glucose disposal mg/kg/min: controls pre-exercise 4.8 ± 1.64; post-exercise 5.79 ± 1.74; *p* < 0.02). Exercise significantly improved lipid induced insulin resistance in both PCOS (glucose disposal mg/kg/min: PCOS pre-exercise 1.15 ± 0.79 vs. post-exercise 1.61 ± 0.75 mg/kg/min, *p* = 0.01), and controls (glucose disposal mg/kg/min: controls pre-exercise 2.51 ± 1.55; post-exercise 3.01 ± 1.35; *p* = 0.01). The glucose disposal rate was significantly compromised in PCOS in comparison with healthy controls for both saline and lipid infusions, independent of the exercise.

The endurance exercise for 8 weeks improved cardiovascular fitness in both PCOS patients (mean VO_2_ max ml/kg/min ± SEM: before 8 weeks exercise, 26.9 ± 1.40; after 8 weeks exercise, 28.7 ± 1.7, *p* = 0.05) and controls (mean VO_2_ max ml/kg/min± SEM: before 8 week exercise, 36.3 ± 2.02; after 8 week exercise, 39.2 ± 1.8; *p* = 0.008). The cardiovascular fitness remained significantly compromised in PCOS in comparison with the controls, even after the exercises driven improvement. Other demographic or biochemical data were not significantly different between the groups after exercise (Table 6).

**Table 6:** Demographic and biochemical changes with exercise between groups.

	**Control**		**PCOS**		**PCOS-control**	
	**Mean (after-before)**	***p*-value**	**Mean (after-before)**	***p*-value**	**Mean difference (95% CI)**	***p*-value**
BMI (kg/m2)	−0.18	0.48	−0.16	0.67	0.06 (0.02, 0.10)	0.97
Waist (cm)	−2.1	8.2 × 10^−3^^*^	−2.0	0.21	−0.10 (−0.22, 0.022)	0.96
TC (mmol/L)	−0.17	0.29	−0.26	0.26	−0.03 (−0.05, 0.01)	0.91
TG (mmol/L)	−0.19	0.02^*^	−0.01	0.84	0.18 (0.16, 0.37)	0.16
HDL-c (mmol/L)	−0.07	0.29	−0.02	0.84	0.05 (0.04, 0.06)	0.54
LDL-c (mmol/L)	−0.06	1	−0.28	0.11	−0.28 (−0.29, −0.27)	0.17
FPG (mmol/L)	−0.04	0.80	0.22	0.12	−0.23 (−0.26, −0.20)	0.66
HOMA-IR	0.58	0.07	−0.25	0.60	−0.83 (−0.86, −0.80)	0.16
VO_2_ max (ml/kg/min)	2.11	8.2 × 10^−3^^*^	2.20	0.05^*^	−12.94 (−13.25, −12.63)	< 0.005^*^

* *Reflect significant value*.

## Discussion

The aim of this study was to determine whether moderate exercise would contribute to the overall improvement in metabolism of PCOS and healthy controls. We found significant differences in the levels of amino acids distinguishing PCOS and control at baseline, which were not present after the exercise, suggesting improvement of the overall metabolism in PCOS individuals. However, while comparing the levels of amino acids before and after exercise within the groups at the baseline there were no differences. The glucose disposal rate and cardiovascular fitness were significantly improved by the exercise.

The comorbidities associated with PCOS include IR, T2D, obesity, and risk of cardiovascular complications (6, 33–35). Those complex diseases manifest alterations in the metabolic profile in comparison with healthy individuals (15, 17, 31); similarly, deregulated metabolism was also observed in women with PCOS (20, 21, 24). The metabolic alterations in the levels of ornithine, phenylalanine, tyrosine, and leucine observed in our study in PCOS at the baseline are in agreement with a previous report (23) further confirming deregulated metabolism in PCOS patients. Increases in the levels of circulating BCAA and AAA were associated with obesity, worse metabolic health followed by future insulin resistance and/or T2D (36). Moreover, BCAA and AAA were recognized as predictors of T2D risk (37), which might potentially contribute to the development of IR via activation of mammalian target of rapamycin complex 1 (mTORC1) and suppression of mitochondrial function in beta-cells (36). Importantly, insulin infusion under euglycemia was shown to decrease levels of BCAA and AAA suggesting a cross-talk between both pathways (38). Thus, elevated levels of BCAA and AAA observed in our study in PCOS suggest their poor metabolic health, which further might lead to IR and T2D. Noteworthy, the elevated levels of BCAA and AAA might potentially reflect compromised GDR, which we observed in PCOS in comparison with healthy control.

We conducted lipid infusions to simulate acute IR and monitor metabolic deregulations under such conditions. Although the lipid infusion significantly altered levels of numbers of acetylcarnitines and lysophospholipides (as we demonstrated on OPLS), the level of BCAA was not affected and among AAA only tryptophan was altered. Elevated levels of long chain carnitines after the lipid infusion might suggest increased fatty acid oxidation required for the energy generation. Furthermore, an elevated level of acetylcarnitine, a product of beta-oxidation of fatty acid further suggests upregulation of this pathway under the lipid infusion. A causative role of tryptophan in fatty acid and triglyceride metabolism was previously reported (39), here we confirm the crosstalk between the pathways and suggest that lipid metabolism is impacting on the tryptophan level in both PCOS and healthy controls.

The improvement in IS in both controls and PCOS after the course of the exercise, which we observed is in agreement with previous studies reporting on exercise-driven improvements in IS in T2D and PCOS (9–11, 13, 40). Moreover, it was shown that the exercise improved the overall glucose homeostasis and lipid metabolism (41, 42) potentially via acceleration of mitochondrial capacity as well as improvement of glucose transport through GLUT4 in muscles (42). In our study, after 8 weeks of exercise the significant differences in the levels of amino acids seen between PCOS and controls at baseline were no longer apparent. The BCAA metabolism contributes to mitochondrial respiration, thus improvement of overall mitochondrial capacity potentially caused by the exercise could further contribute to the normalization of BCAA metabolism in our study. However, we did not observed a significant difference in metabolic levels within the group (PCOS, control) after testing for significance before and after exercise. This could be due the small group of individuals enrolled for this study, given the metabolic heterogeneity, previously reported in women with PCOS (20). Because moderate exercise could contribute to the overall improvement of body metabolism, as the PCOS amino acid profile did not differ to control, a further study with a larger number of individuals would delineate the interplay between moderate exercises, IR and amino acid metabolism.

Exercise increased VO2 max and increased IS as expected, with an exaggeration of IR following the acute lipid infusion indicating that the lipid infusion technique may be of value to modulate insulin resistance directly to assess its metabolic impact, rather than the more common situation of interventions to reduce IR therapeutically. The acute increase in IR from the lipid infusion likely results from an acute rise in diacylglycerol (DAG) in the muscle that subsequently induces insulin resistance by reducing non-oxidative glucose disposal through PKCθ activation (25). Following exercise, the improvement in IS was likely due to increased fat oxidation with moderate intensity exercise (40), and a reduction in intra-myocellular fat metabolites would improve IR (13); notably, IS did not reverse entirely to that of the control subjects, indicative of the underlying metabolic defect in PCOS (35). However, this study suggests that PCOS women who undertake moderate intensity exercise for 180 min per week without weight loss may address their metabolic differences and reduce their IR.

The strengths of this study were using gold standard methodology for this intensive interventional study, the well supervised exercise intervention and the state-of-the-art metabolomics analysis available to us. The limitations were that this was a small group of women with PCOS, though all fulfilled all three of the diagnostic criteria for its diagnosis thereby reducing heterogeneity, and the groups were not well matched for BMI and age.

In summary, the baseline amino acid profile in PCOS reflected that seen in obese non-PCOS subjects and was reversed to that seen in controls by exercise without a change in weight, in association with an increase in IS. This shows that exercise may normalize the amino acid metabolome irrespective of weight. Nevertheless, a study focusing on a larger number of individuals would be required to provide further insights in PCOS metabolism in the context of physical activity.

## Precis

Branched chain amino acids and aromatic amino acids measured using targeted metabolomics approach showed differences at baseline in PCOS that normalized following an exercise program that improved insulin sensitivity; they did not differ following maximal insulin resistance stimulation with a lipid infusion before and 8 weeks after exercise. The exercise-driven improvement in metabolism corresponded with improved in insulin sensitivity and cardiovascular fitness.

## Ethics Statement

All patients provided written informed consent to participate in the study, which was conducted in accordance with the Declaration of Helsinki and Good Clinical Practice. This study was approved by the Leeds Central Research Ethics Committee, Yorkshire and Humber REC no. 10/H1313/44.

## Author Contributions

SA devised the study that was undertaken by MA. MK and AH conducted the measurements. AH and KS were responsible for the metabolic analysis and SD undertook the statistical analysis. All authors contributed to the writing of the manuscript.

### Conflict of Interest Statement

The authors declare that the research was conducted in the absence of any commercial or financial relationships that could be construed as a potential conflict of interest.

## Supplementary Material

Supplementary Figure 1 Lipid infusion causes general metabolic changes in PCOS and healthy controls. **(A)** OPLS reveals challenge (saline/lipid) and time-dependent separation between groups. **(B)** OPLS loading plot shows contribution of different metabolites to the separation.

Supplementary Figure 2 No separation between PCOS and healthy controls based on 163 measured metabolites.

Supplementary Table 1 Impact of lipid infusion on amino acid metabolism.

Supplementary Table 2 Effect of exercise on intralipid-induced insulin resistance within groups.
