# Supplementary figures and images for: Metabolomics of Dynamic Changes in Insulin Resistance Before and After Exercise in PCOS
Source: Front Endocrinol (Lausanne). 2019 Feb 27;10:116. doi: 10.3389/fendo.2019.00116 (PMC6400834; doi:10.3389/fendo.2019.00116)

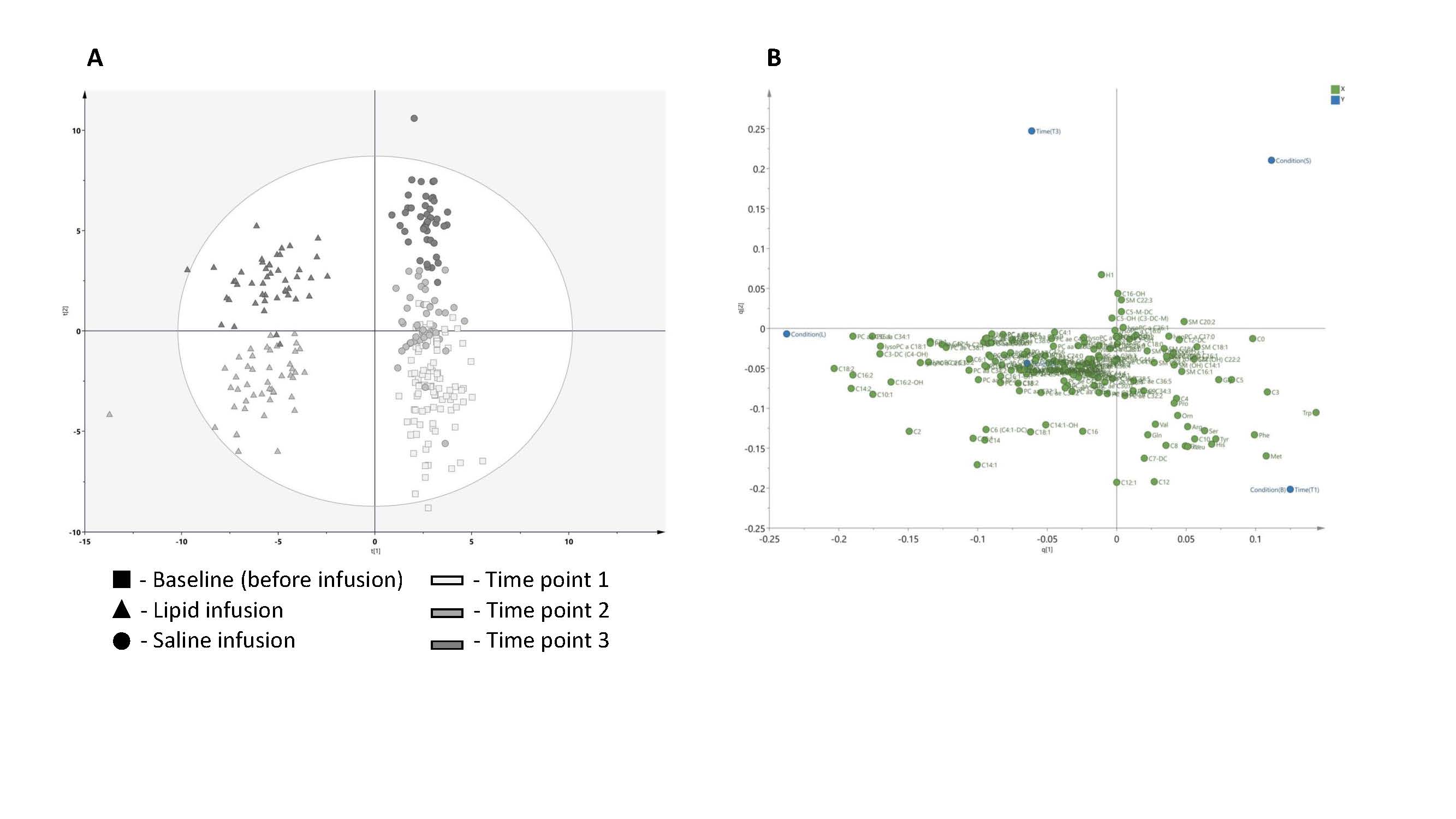

Supplement: Supplementary Figure 1 — Lipid infusion causes general metabolic changes in PCOS and healthy controls. (A) OPLS reveals challenge (saline/lipid) and time-dependent separation between groups. (B) OPLS loading plot shows contribution of different metabolites to the separation. [file Image_1.JPEG]

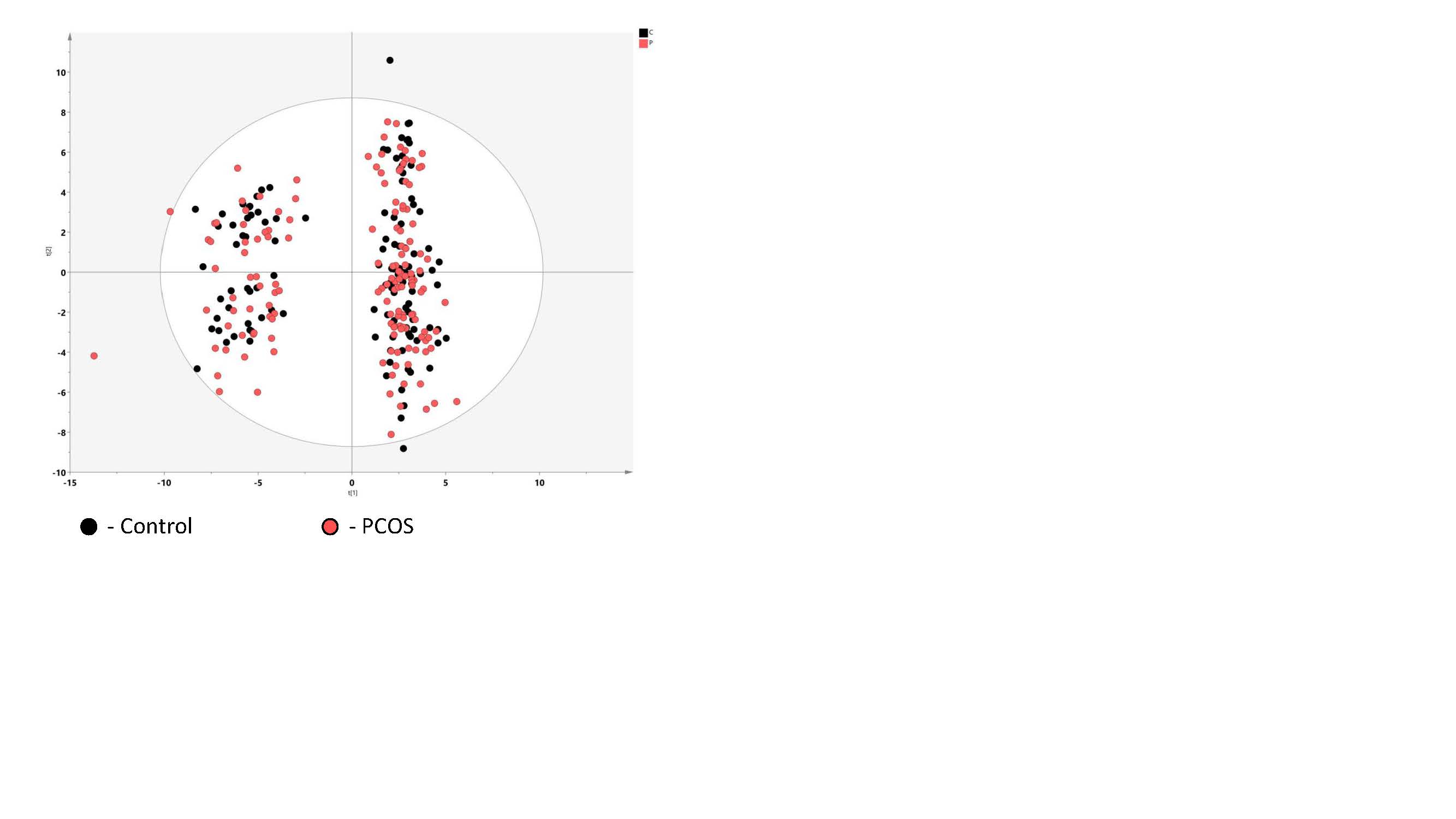

Supplement: Supplementary Figure 2 — No separation between PCOS and healthy controls based on 163 measured metabolites. [file Image_2.JPEG]
