# Supplementary material for: Metabolomics of Dynamic Changes in Insulin Resistance Before and After Exercise in PCOS
Source: Front Endocrinol (Lausanne). 2019 Feb 27;10:116. doi: 10.3389/fendo.2019.00116 (PMC6400834; doi:10.3389/fendo.2019.00116)
Supplement: Supplementary Table 2 — Effect of exercise on intralipid-induced insulin resistance within groups. [file Table_2.DOCX]

|  | **Control** | | | **PCOS** | | |
| --- | --- | --- | --- | --- | --- | --- |
|  | **Glucose disposal mg/kg/min** | |  | **Glucose disposal mg/kg/min** | |  |
| **Infussion** | **Before exercise** | **After exercise** | **p-value before vs. after exercise** | **Before exercise** | **After exercise** | **p-value before vs. after exercise** |
| Saline | 4.80 (1.65) | 5.79 (1.74) | 0.02 | 3.25 (0.76) | 3.95 (1.45) | 0.13 |
| Lipid | 2.51 (1.55) | 3.01 (1.35) | 0.03 | 1.15 (0.79) | 1.61 (0.75) | 0.01 |
| **p-value saline vs. lipid** | 5.50x10^-4^ | 4.97x10^-4^ |  | 1.99x10^-7^ | 2.89x10^-4^ |  |
